# Contrast and Homogeneity Feature Analysis for Classifying Tremor Levels in Parkinson’s Disease Patients

**DOI:** 10.3390/s19092072

**Published:** 2019-05-04

**Authors:** Guillermina Vivar, Dora-Luz Almanza-Ojeda, Irene Cheng, Juan Carlos Gomez, J. A. Andrade-Lucio, Mario-Alberto Ibarra-Manzano

**Affiliations:** 1 Department of Electronics Engineering, Universidad de Guanajuato, Salamanca Gto. C.P. 36885, Mexico; g.vivarestudillo@ugto.mx (G.V.); dora.almanza@ugto.mx (D.-L.A.-O.); jc.gomez@ugto.mx (J.C.G.); andrade@ugto.mx (J.A.A.-L.); 2 Department of Computing Science, University of Alberta, Edmonton, AB T6G 2E9, Canada; locheng@ualberta.ca

**Keywords:** tremor, Parkinson’s disease, SDH method, classification

## Abstract

Early detection of different levels of tremors helps to obtain a more accurate diagnosis of Parkinson’s disease and to increase the therapy options for a better quality of life for patients. This work proposes a non-invasive strategy to measure the severity of tremors with the aim of diagnosing one of the first three levels of Parkinson’s disease by the Unified Parkinson’s Disease Rating Scale (UPDRS). A tremor being an involuntary motion that mainly appears in the hands; the dataset is acquired using a leap motion controller that measures 3D coordinates of each finger and the palmar region. Texture features are computed using sum and difference of histograms (SDH) to characterize the dataset, varying the window size; however, only the most fundamental elements are used in the classification stage. A machine learning classifier provides the final classification results of the tremor level. The effectiveness of our approach is obtained by a set of performance metrics, which are also used to show a comparison between different proposed designs.

## 1. Introduction

Parkinson’s disease (PD) is a neurodegenerative disorder of unknown pathogenesis characterized by tremors, rigidity, and slowness of movements. This disease is considered the second most common disorder after Alzheimer’s disease [1] and is associated with progressive neuronal loss of the *substantia nigra* and other brain structures [2]. In the most populated countries, PD patients over the age of 50 were between 4.1 and 4.6 million in 2005 and it is estimated that it could reach around 9 million by 2030 [3].

Tremors are the most common symptom of Parkinson’s, which is a clinical characteristic that allows the diagnosis of some degenerative diseases. The detection and assessment of tremors have become an important aspect of PD because it is the reference of severity that allows the planning of treatment, by estimating how it affects the daily life of a patient [4]. Tremor severity is typically quantified using clinical metrics, in which the subject’s condition and their ability to perform motor skills are evaluated [5]. The most commonly used PD metrics are the Hoehn and Yahr (H&Y) and the Unified Parkinson’s Disease Rating Scale (UPDRS) [6]. The Hoehn and Yahr scale (H&Y) detects the stages of the disease [5]. The Unified Parkinson’s Disease Rating Scale (UPDRS) is one of the primary scales used for assessing tremors due to its reliability and validity [7]. The scores in UPDRS Part III relate to motor examination and indicate ranges of levels between 0 to 4, with the following correspondence: 0 to normal, 1 to slight, 2 to mild, 3 to moderate, and 4 to severe. The correct classification of the tremor severity provides the means for checking disease progression and the corresponding treatment. Thus, a revised version of the UPDRS is the Movement Disorder Society’s UPDR scale (called MDS-UPDRS) proposed for improving identification of the levels of PD [7]. This updated version provides the most explicit instructions to perform the tests and a greater number of PD symptoms, such as nonmotor [8].

Usually, during the test for PD diagnosis, the neurologist observes the patient while they perform motricity tasks, then the final score is given using the UPDRS [9]. It is important to note that the diagnosis of the tremor level in hands using any scale is subjective and is based on neurologist clinical experience [10], being prone to human errors. The studies reveal that ∼25% of diagnoses are incorrect; consequently, the treatment is too [2]. For this reason, early prognosis and objective assessments of the tremor level in Parkinson’s disease could be more appropriate by using automatic strategies and devices that assist medical diagnosis.

Many studies using technology tools for detecting early symptoms of PD have been proposed in [11,12]; however, this technology needs to be attached to the body to acquire the motion information of the tremor that becomes uncomfortable for patient performance. In contrast, commercial wireless devices have demonstrated high reliability and accuracy during hand position detection and tracking. That is the case of the leap motion controller (LMC), which has been used to track hand movements for different applications [13,14,15]. 

In this work, the main idea is detecting the severity of tremor in the first two stages of Parkinson’s disease by characterizing hand behavior when patients perform gentle exercises. In [16], a previous version of this work proposed the detection of Parkinson’s disease from a patient’s database, showing only two classes: (1) with and (2) without Parkinson’s disease (PD). In this work, we extend the classification to three categories: 0—normal, 1—slight, and 2—mild using an LMC device. In the previous work, classes 1 and 2 were considered in one class, facilitating the characterization. However, these classes represent the two initial levels of PD; therefore, to have a more accurate diagnosis depends on detecting both categories separately. Besides, the tremor pattern between classes 0 and 1 is similar, due to level 1 representing the earliest low-intensity tremor, hard to see but manifested in patients. To overcome this, a detailed analysis of nine texture features was performed to find the most discriminant features for the new classification scheme. In the previous work, we concluded that the highest classification accuracy was obtained using the bagged tree (BgT) and the Fine-K Nearest Neighbor (F-KNN) classifiers. Based on that result, we use here both classifiers, to perform an in-depth analysis of each texture feature varying the window size, with the contrast and the homogeneity being the most accurate. We have evaluated the performance of the classifier varying the window size and compared with similar works proposed in the literature. Furthermore, this proposed approach intends to minimize the discomfort of aging people during clinical evaluations by providing a non-wearable and portable sensor that detects the level of tremor and monitors its progression using a graphical user interface.

## 2. Background 

Movement disorders are neurologic syndromes characterized by an excess of motion or a paucity of voluntary and automatic movements, unrelated to weakness or spasticity [17]. Movements can be categorized as automatic, voluntary, semi-voluntary, and involuntary. Patients of Parkinson’s suffer different main motor symptoms—bradykinesia (slowness of action), rigidity, and tremor [18]. Fahn et al. [19] define tremors as involuntary oscillations of any part of the body around any plane; while Elble [20] describes tremors as a neurologic sign that is produced by many neurologic and systemic diseases. The study of tremors in patients with Parkinson’s disease (PD) have focused on measuring characteristics such as amplitude, frequency components, and quantified data using statistical methods; this characterization provides useful clinical information for the diagnosis and monitoring of tremors [21,22]. 

**Figure 1:**
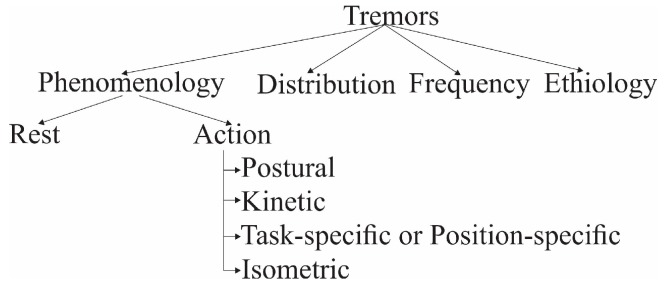
Tremor classification.

Tremors can be classified according to their phenomenology, distribution, frequency, or etiology [20,23,24,25]. Phenomenologically, tremors are divided into two major categories—rest tremors and action tremors [26]. Action tremors occur with the voluntary contraction of muscles, and they can be subdivided into postural, kinetic, task-specific or position-specific, and isometric, as shown in Figure 1. Postural tremor is when a distal limb stands against gravity. Kinetic tremor can be seen when the voluntary movements start, while the limb is in motion, and affects body parts as it approaches the target, for instance, finger to nose. Position-specific tremors occur while the patient stands in a particular posture. Depending on the neurologist’s observations, different tasks are chosen to assess the severity of tremor.

In the past years, several methods have been developed to measure and analyze characteristics of patients with PD using technology-based objective measures (TOMs). Oung et al. in [11] presented state-of-the-art methods in early detection and monitoring PD symptoms through technological tools. These tools were categorized into five groups: (1) electromyography (EMG), (2) electroencephalogram (EEG), (3) computed tomography (CT) scans or magnetic resonance imaging (MRI), (4) unimodal wearable sensors (gyroscopes, accelerometers), and (5) audio sensors. They concluded that the EEG can precisely identify brain activity, but it can be uncomfortable to most patients during data retrieval. Moreover, the measurements are unreliable if the patient had been medicated before the test. Like the MRI method, patients need to stay immobile for some period during the scanning in an enclosed space. Also, it requires an experienced radiologist to interpret the images which increases the overall cost. On the other hand, CT scans insert a dose of radiation into the patients. Regarding wearable sensors, their success directly depends on the sensor’s performance, cost, and reliability. Finally, speech signals are a non-invasive technique for telemonitoring in medical care but unreliable for tremor level detection in early stages.

In [12], the authors describe several methods to provide an objective assessment of PD detection, progression, and evaluation of treatment, covering different motor and nonmotor aspects of PD. In the motor aspects, the most common devices used are IMUs (inertial measurement units) composed by accelerometers and gyroscopes, such as in [27]; followed by smartphones, such as in [28]. In general, the sensitivity and specificity reported for tremor classification is higher than 80%. The classification of PD employs machine learning techniques providing an effective alternative for diagnosis using different types of sensors [29,30,31,32,33,34]. Several studies have explored wearable sensors to detect motor disorders in PD [27,35,36] but as we mentioned above by Oung [11], the patients must be wearing the measuring device, i.e., for assessment of tremors in hands, the sensors are used or attached, for instance, to the wrist or finger, such as in [37,38]. Thus, in the present study, we focus on a non-invasive sensor to capture the movements of the hands. Leap motion has been used in other research, for instance, in [13] leap motion is used to measure bradykinesia in patients with PD. Similarly, in [39] the study of the authors confirm that is possible to measure a tremor without a wearable sensor.

The primary challenge is to provide a non-invasive and economical approach to diagnose, monitor, and classify the severity of tremors during routine clinical visits in early stages of patients with PD. Also, we look to provide similar results to a neurology evaluation based on the MDS-UPDRS using a machine learning classifier and leap motion control.

## 3. Materials and Methods

### 3.1. Methodology 

**Figure 2:**
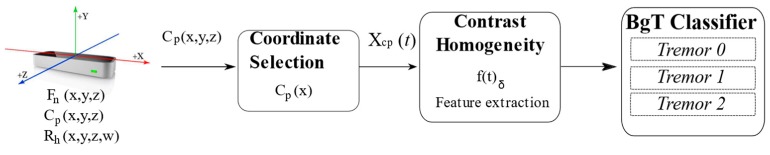
Block diagram of the analysis of tremors for Parkinson’s classification. BgT: bagged tree.

The strategy proposed for detecting a tremor in patients with Parkinson’s disease is shown in Figure 2. It consists of the following general stages: (1) data acquisition, (2) coordinate selection, (3) feature computation, (4) classification in three levels.

### 3.2. Data Acquisition 

The proposed methodology uses data acquired from a leap motion controller (LMC). The LMC is an interactive device used primarily for hand gestures and finger position detection [40], which includes three infrared light (IR) emitters and two IR cameras used as depth sensors. It provides a gesture and position tracking system with sub-millimeter accuracy in a field of view of ~150°. The user´s hand is captured between 25 to 600 mm above the sensor.

The database used in this work is the same as [41], and it has been acquired during the exercises performed by twenty patients diagnosed with PD as a part of their rehabilitation routines. The study was conducted at the Edmonton Kaye Alberta Clinic. Each patient provided prior informed consent to perform the tests. The data was collected for both right and left hands for 11 males and 9 females with a mean age of 69 years old and a standard deviation of 14.

#### 3.2.1. Experimental Protocol

**Figure 3:**
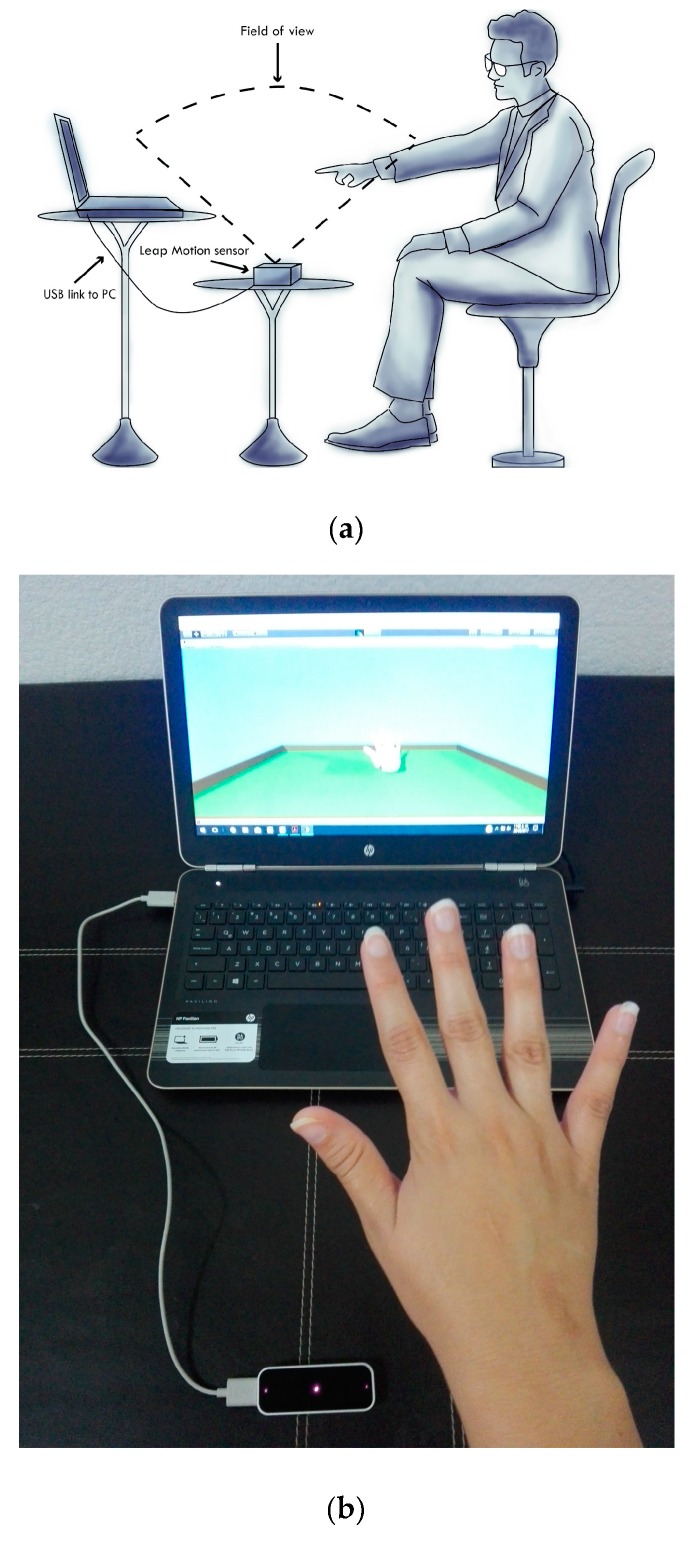
Experimental tests. (**a**) Scheme showing the position of the patients and the elements to acquire the signal. (**b**) View of the patients when they perform the task using a natural interaction system through a virtual reality interface.

The experimental tests consisted of the use of a natural interaction system as a medical tool, through a virtual reality interface. Concurrently, the measurement of postural and kinetic tremor was acquired according to the MDS-UPDRS standard. The resulting data from this natural interaction between patients and virtual reality was acquired using the LMC [41]. Individually, the signal was registered once each patient was comfortably seated in a chair in front of the sensor as shown in Figure 3a. First, in the experiment, the patient extended the right hand with the wrist straight and the fingers comfortably separated so that they did no touch each other for 10 s in the initial position. This exercise was indicated on the screen by a red sphere, as shown in Figure 3b, and the patient tried to move or push the ball located in the center of the scene, to the final place positioned on the right following a straight path. Then, the same activity is repeated with the left hand, but now the last area is on the left side. Figure 3b illustrates the task performed by the patients in the experiment using the virtual reality interface. The duration of the test was established at 15 s because the main idea was to induce the patient into a stressed situation or concentration and to monitor how they focused when performing a specific activity in daily life. The acquired data is illustrated in Table 1 and is divided by tremor levels groups, 0—normal, 1—slight, and 2—mild, by gender, and by hand. For the test, the LMC was set up to work at a rate of 40 samples per second (sps). The database contained one patient with tremor level 3 in the right hand. We considered this data as an outlier and ignored it. Therefore, the total samples used for the experimental tests were 39. Moreover, the database contained patients with different or unequal levels of tremor by hand. 

#### 3.2.2. Tremor Database

The database acquired is a collection of 600 samples per hand of the volunteers that includes: position and velocity of the fingertips *F_n_* (*x, y, z*), the palm center *C_p_* (*x, y, z*), and the rotation of the hand *R_h_* (*x, y, z, w*). Each acquired sample corresponds to 40 variables in the 3D space, as shown in Table 2.

As we mentioned above, the hand motion in patients with PD was spontaneous oscillations. Thus, we analyzed the tremor as a temporal change in the palmar region position concerning the LMC reference system. 

### 3.3. Feature Extraction 

In [42], a feature is defined by a function that quantifies the significant properties of an object or a signal. The most common element used for data characterization and description is texture, which provides the spatial relation from an individual data point and its neighbors. Moreover, texture provides the estimation of properties between two or more data points at near locations, represented as scalar numbers, discrete histograms, or empirical distributions. The sum and difference of histograms (SDH) technique estimates the texture features in a window [43]. The SDH requires basic arithmetic operations and less memory storage in comparison with other texture techniques. Furthermore, it stores relevant information about the image content. In this work, we adopt the SDH method used in [44,45] for calculating texture features from signals. As we mentioned above, SDH computes histograms that collect results of the addition and subtraction of images. For this propose, we adopt the windowed scheme of SDH and calculate histograms from a 1D data vector in a neighborhood. Thus, SDH computes the addition and subtraction of data positions delimited by a window size shifted through the whole dataset in one dimension, instead of pixels on an image. Hence, SDH shows the distribution of the motion by the spatial relation between a data point and its neighbors, where each point represents the position of the palmar region concerning the LMC.

**Figure 4:**
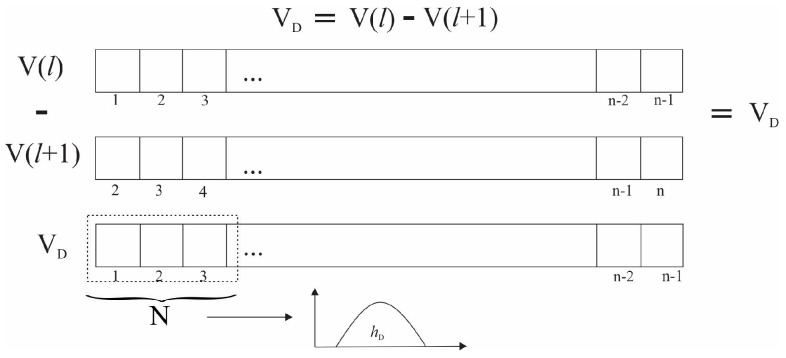
General process of the sum and difference of histograms (SDH) method in a vector.

For simplicity, we adapt the spatial domain I (k, l) to the vector space V(*l*) in the time domain. In this approach, the V(*l*) vector is composed by *n* samples of the *C_p_*(*x*) positions, acquired at 40 sps for 15 s and (*l* ϵ [1, *n*]) where *n* = 600. The difference vector (*V_D_*) is obtained by subtracting each data point and its neighbor, separated by a displacement (*M*), in this case *M* = 1, i.e., the previous sample. Afterward, we specify the region D as a subset of indexes defined by the window size *N* in which the texture features will be calculated, where *N = Card{D}*. The difference histogram (*h_D_*) is calculated from (*V_D_*). In this case, *V_D_* represents the number of times that *x* position appears in the difference vector over the domain D. Figure 4 shows the process to obtain *V_D_* and *h_D_*. Similarly, the sum vector (V_S_) and the sum of the histogram (*h_S_*) is calculated. 

Finally, the normalized difference histogram $\hat{h_{D}(}l)$ is given by Equation (1).

(1) $$\hat{h_{D}(}l)=\frac{h_{D}\left(l\right)}{N}$$

Similarly, the normalized sum histogram $\hat{h_{S}(}l)$ was calculated. Afterward, nine texture features were computed using the adequation proposed in [43] from the SDH, mean, variance, energy, correlation, entropy, contrast, homogeneity, cluster shade, and cluster prominence. 

### 3.4. Coordinate Selection

We analyzed the tremor as a temporal change in the position of the hand concerning the LMC reference system by tracking the palm center *C_p_* (*x, y, z*) position. Table 3 lists the different machine learning methods used during the first stage of the experimental tests, to classify texture features obtained by the SDH technique for each coordinate of *C_p_*. A comparative analysis was made between the classifications obtained using each coordinate *C_p_*(*x*), *C_p_*(*y*), and *C_p_*(*z*) independently, and the xyz position of the palmar region *C_p_* (*x, y, z*) for feature extraction. The accuracy results for a window size of 125 are illustrated in Table 3.

On average, the accuracy obtained for all classifiers achieved the highest value for coordinate x. Then, the use of the x coordinate of the palmar region *C_p_*(*x*) highly reduced the amount of data to be processed and allowed us to analyze only the motion of the right and left hand in time (C*_p_*(*x*)). 

### 3.5. Select the Feature

In our previous work [16], different supervised machine learning methods were tested, such as k-nearest neighbors, support vector machines, decision tree, linear discriminant, and an ensemble of classifiers to classify texture features obtained by the SDH method in different tremor levels. The results were compared with other approaches for tremor signal classification; the highest performance results were achieved by bagged tree (BgT) and fine KNN (F-KNN), for two classes—with or without PD. 

The bagged tree (BgT) is a classification algorithm that trains a set of weak classifiers by replicating the training set; the outputs of these classifiers are combined, and the final class is obtained by vote [46]. On the other hand, the k-nearest neighbor is a density estimation method which compares the distance between a weighted-features vector and each set into the feature space of the training classes [47]. Both machine learning algorithms improve the stability and accuracy of PD level detection using statistical features [48].

**Figure 5:**
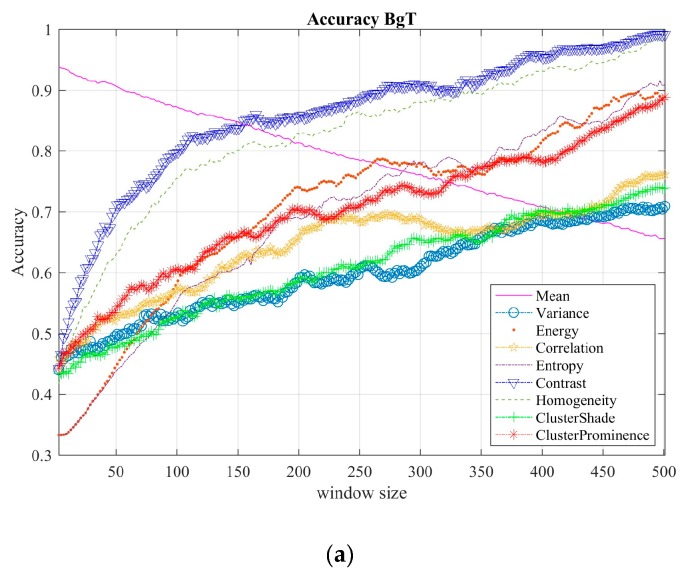

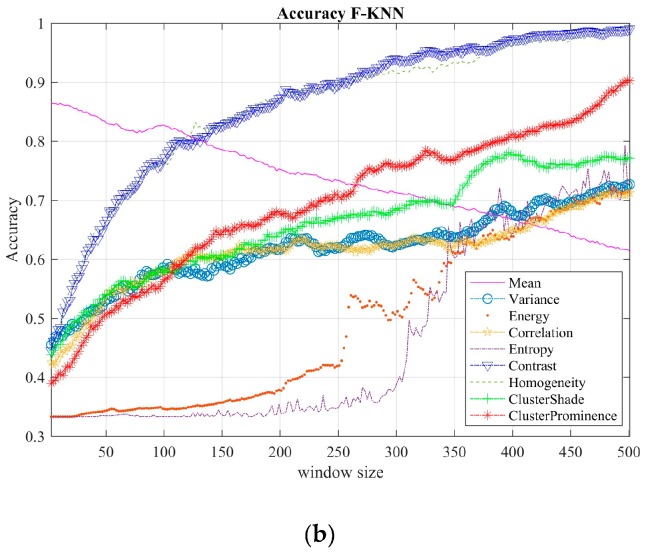
Comparison of the classification results for different texture features. (**a**) For the BgT classifier and (**b**) for the F-KNN classifier.

Thus, feature selection consists of testing both classifiers for each texture feature mean, variance, correlation, contrast, homogeneity, cluster shade, and cluster prominence using different window sizes (from 3 to 501). Plots in Figure 5 show the performance comparisons among the texture features, using the bagged tree, as shown in Figure 5a, and F-KNN classifier, as shown in Figure 5b. 

Note that contrast and homogeneity texture features, defined by Equations (2) and (3), are the most representative for tremor level classification. The homogeneity measures the closeness to the displacement distribution of the signal; likewise, contrast measures the local changes in the palmar region positions (*C_p_*(*x*)). In other words, contrast quantifies the discontinuity of *x*-coordinate displacements of *C_p_*(*x*), while homogeneity measures the similarity of *x*-coordinate displacements of *C_p_*(*x*). Thus, both features were used to characterize the dataset during the classification stage.

(2) $$Contrast=\underset{l}{\sum }l^{2}\cdot \hat{V_{D}}\left(l\right)$$

(3) $$Homogeneity=\underset{l}{\sum }\frac{1}{1+l}\cdot \hat{V_{D}}\left(l\right)$$

### 3.6. Tremor Classification and Performance

**Figure 6:**
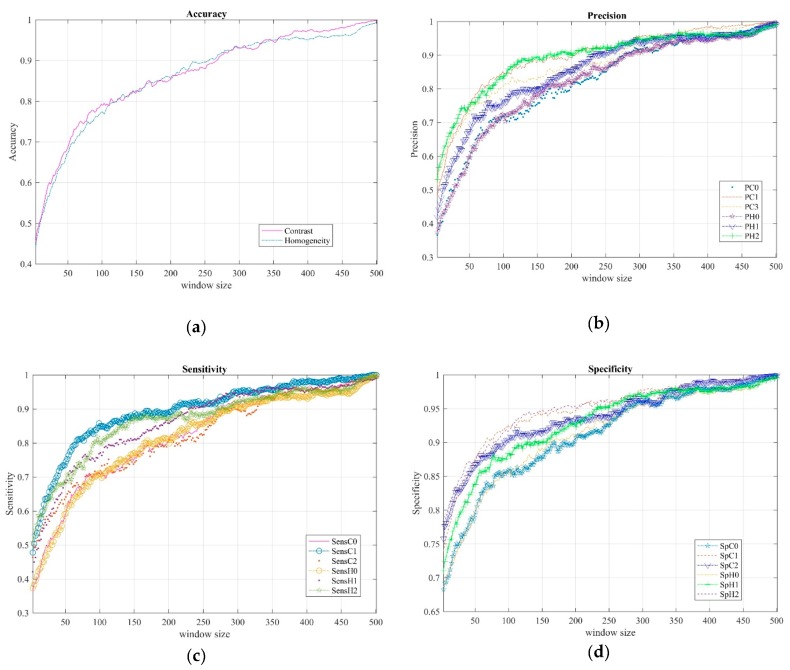
Performance metrics of a BgT classifier for different window sizes. (**a**) Accuracy of contrast (C) and homogeneity (H), (**b**) precision (P), (**c**) sensitivity (Sens), and (**d**) specificity (Sp) of contrast (C) and homogeneity (H) for each class (0, 1, and 2), respectively.

Supervised machine learning based on statistical classification is one of the most common techniques to design intelligent systems from an input dataset. The final classification results were carried out using a bagged tree classifier for contrast and homogeneity features as presented in the previous section. We acquired our measurements from 21 patients, following the experimental protocol described above. Moreover, we considered as a ground-truth set the diagnosis provided by clinicians, where for each patient data was previously labeled as: normal (0), slight (1), and mild (2). The metric values in Figure 6 were obtained by classifying 5400 samples in total; that is, 1800 per class. Left and right hand coordinates for the three classes were combined and balanced. The experimental tests were repeated 30 times for each window size.

The parameters used to compute homogeneity and contrast were: (i) displacement *M* was set to 1, (ii) x-coordinate of the hand from patients diagnosed with different Parkinson’s levels, (iii) 250 different window sizes in the range of 3 to 501. For each window size the classification was repeated 30 times. All the experiments relied on the BgT classifier programmed using the toolbox of MATLAB R2016b. We want to point out that the number of total samples *N* used during classification depends on the window size. As different window sizes were tested, the variability of *N* in our experiments did not affect the training and validation stages because we used 70% and 30% of *N* samples, respectively, in each stage.

Plots a–d in Figure 6 show the accuracy (Acc), precision (P), sensitivity (Sens), and specificity (Sp), respectively, for contrast and homogeneity features as the window size increases. Note that metric values improved as window size increased for both features. In the next section, we show these metrics for only three window sizes to illustrate specific classification results in detail. 

## 4. Results and Discussion

In this section, we present a more detailed analysis of the classification results presented in the previous section and a comparison of our proposed approach with similar approaches in the literature. 

Table 4 illustrates the classification results carried out for three specific window sizes—149, 299, and 449; these values represent the three first quartiles in the range of window sizes (abbreviated as *ws* in the table). For each metric, we show minimal, averaged, and maximal value to describe the metric behavior during classification. These results were obtained using a bagged tree classifier. The first two rows of the table show the accuracy for contrast and homogeneity features, respectively. Note that the homogeneity feature for *ws* = 149 throws better accuracy values than contrast, unlike contrast which produces better accuracy for *ws* = 449. However, the difference between both elements for accuracy cannot reach more than 0.68%, nor the rest of the metrics show significant differences. Moreover, precision, using the contrast feature, achieves better percentage results for tremor level 1 than homogeneity, while homogeneity is better for classifying tremor level 2, regardless the window size. Similarly, the sensitivity and specificity metrics show that the contrast feature is better for classifying tremor level 1, whereas homogeneity classifies tremor level 2 better.

Notice that in the case of level 0, all the metrics throw lower values than levels 1 and 2. This case happens because level 0 represents a normal condition (without a tremor in patients with PD), then the feature space is low discriminant at this level, being more difficult to establish with a reference, and it can be misclassified easier than levels 1 or 2. The obtained results show that our proposed method classifies the patient measurements following MDS-UPDRS (see Section 3 in the scale) in tremor levels 0, 1, and 2 with accuracy above 98% and 95% for contrast and homogeneity, respectively. 

In the rest of this section, we compare the performance of this approach and the existing methods that classify three levels of tremor in patients with PD based on machine learning. In Table 5, we summarize the performance comparison between similar approaches and the results of the proposed work. The first column enlists the different studies in the literature; the second column indicates the technology used to retrieve data; in the third to fifth columns we report the accuracy, specificity, and sensitivity metrics. The rest of the columns show, respectively, the classifier employed, the standard to assess tremor patients and the levels classified (five in some cases).

Notice that our approach reaches better accuracy measures than [37,38,49,50]. These approaches classify four or five tremor levels and our approach classifies only three levels. However, these approaches have in common that they use wearable sensors to acquire measurements which are unsuitable for most of the patients. Furthermore, their evaluation rules are based on UPDRS, while the MDS-UPDRS is an updated version. Meanwhile, Bazgir et al. [51] employ a naïve Bayes classifier reaching a perfect accuracy performance, although a smartphone must be attached to the patient during data retrieval. Also, the authors in the same work propose the implementation of an Advanced RISC Machine (ARM)-microcontroller to retrieve data, but this device reaches lower accuracy value (94%) than our approach.

## 5. Conclusions

This work presents a strategy based on SDHs for the classification of three levels of tremor employing homogeneity and contrast features. This approach was developed as an alternative to a non-invasive and automatic system for the diagnosis and monitoring of tremor level in patients with PD. By analyzing the provided metrics, we found reliable classification results for diagnosing Parkinson’s disease in the early stages. Specifically, the highest scores were obtained to levels 1 and 2. This result is expected because level 0 represents the lack of malign tremor, thus, the classification error increases. Our results show that it is possible to classify the different levels of tremor in patients with Parkinson’s disease using only two statistical features, such as homogeneity and contrast. Furthermore, the proposed classification approach is independent of the hand used by patients. We used the LMC which is a cheap, non-invasive, portable device to acquire accurate signal data. Moreover, the natural interaction with the leap motion and the patient helped the patient with the discomfort in clinical evaluations. Finally, performance metrics demonstrate that our proposed approach can be used for diagnosing Parkinson’s disease in the early stages.

The possible applications of this method are for helping clinicians in diagnosis or for monitoring patient performance during rehabilitation routines. So far, this work consists of an unwearable sensor (LMC) and of a graphical user interface (GUI) that displays the retrieved data position of the hand and serves as a reference for patients during the activity. GUI and LMC drivers run on a personal computer and the experimental test is carried out with patient sitting down in front of the machine. However, further research includes implementing the GUI and the methodology on a reconfigurable device, which is smaller and portable compared with the CPU of a PC, to provide a mobile device used at home by patients for monitoring progression of PD.

## Figures and Tables

**Table 1:** Distribution of patients in different tremor level, gender, and the hand used.

Tremor Level	Gender (F—Female/M—Male)	Right Hand	Left Hand	Subtotal
0—Normal	F	7	6	13
	M	5	4	9
1—Slight	F	2	3	5
	M	4	5	9
2—Mild	F	0	0	0
	M	1	2	3
Total		19	20	39

**Table 2:** Hand measurement captured with the leap motion controller (LMC).

Data	Acquired Data	Coordinates	Total
Position, velocity	Thumb, index, middle, ring, little, palmar region	(x, y, z)	36
Rotation	Hand	(x, y, z, w)	4
**Total**			40

**Table 3:** Classification performance among single coordinate position and the xyz position. This accuracy is obtained for a window size of 125.

Classifier/Acc.	*C_p_*(*x*)	*C_p_*(*y*)	*C_p_*(*z*)	*C_p_* (*x, y, z*)
Bagged Tree	100	100	100	100
Boosted Tree	99.8	33.3	99.5	100
Coarse Gaussian Support Vector Machine (SVM)	73.1	78.3	63.5	52.3
Coarse K-Nearest Neighbor (KNN)	90.4	96.8	90.4	86.5
Cosine KNN	99.6	99.9	100	99.5
Cubic SVM	99.8	93	86.8	92.5
Complex Tree	99.7	99.9	99.4	99.8
Cubic KNN	99.6	99.8	96.6	99.3
Fine Gaussian SVM	99.8	93	82.9	92.6
Fine KNN	100	100	99.4	100
Linear Discriminant	62.1	61.1	60	53.5
Linear SVM	67.3	77.8	57.3	52.9
Medium Gaussian SVM	97.1	93	77	87.6
Medium KNN	99.6	99.8	97.2	99.4
Medium Tree	99.7	99.9	93.7	96.3
Quadratic Discriminant	49.5	61.5	46.2	43.6
Quadratic SVM	99	98.2	81.3	85
Random Under Sampling (RUS) Boosted Tree	99.7	33.3	93.7	96.3
Subspace KNN	99.6	99.4	94	99.3
Subspace Discriminant	67.5	63.6	62.8	51.5
Simple Tree	88.3	97.2	77.8	74.5
Weighted KNN	100	99.9	98.7	99.9
Mean Accuracy	90.50	85.39	84.46	84.65

**Table 4:** Detailed statistical results of tremor level classification: accuracy (Acc), precision (P), sensitivity (Sens), and specificity (SP), using window sizes (ws) of 149, 299, and 449 for calculating contrast (C) and homogeneity (H) features.

ws	149			299			449		
	Min	Mean	Max	Min	Mean	Max	Min	Mean	Max
**Acc C**	0.8049	0.8189	0.8286	0.9181	0.9300	**0.9395**	**0.9681**	**0.9801**	**0.9859**
**Acc H**	**0.8057**	**0.8257**	**0.8407**	**0.9226**	**0.9311**	**0.9395**	0.9427	0.9599	0.9710
P C0	0.7195	0.7533	0.7880	0.8879	0.9159	0.9395	0.9487	0.9714	0.9872
P C1	**0.8407**	**0.8771**	**0.9006**	**0.9324**	**0.9546**	**0.9680**	**0.9724**	**0.9880**	**0.9951**
P C2	0.7979	0.8262	0.8529	0.8879	0.9195	0.9386	0.9566	0.9811	0.9921
P H0	0.7437	0.7835	0.8084	0.8701	0.9063	0.9297	0.9230	0.9514	0.9724
P H1	0.7646	0.7996	0.8302	0.9208	0.9367	0.9520	**0.9487**	0.9628	0.9763
P H2	**0.8577**	**0.8939**	**0.9143**	**0.9279**	**0.9505**	**0.9689**	0.9398	**0.9655**	**0.9822**
Sens C0	0.7175	0.7480	0.7731	0.8879	0.9148	0.9416	0.939	0.9693	0.9831
Sens C1	**0.8500**	**0.8786**	**0.8962**	**0.9203**	**0.9470**	**0.9718**	**0.9765**	**0.9901**	**0.9970**
Sens C2	0.6983	0.7379	0.7702	0.8419	0.8991	0.9172	0.9436	0.9634	0.9820
Sens H0	0.7280	0.7693	0.7898	0.8914	0.9092	0.9263	0.9229	0.9503	0.9725
Sens H1	0.7935	0.8095	0.8399	**0.9152**	**0.9383**	**0.9666**	**0.9241**	**0.9646**	**0.9826**
Sens H2	**0.8237**	**0.8782**	**0.9046**	0.8915	0.9177	0.9517	0.9003	0.9503	0.9798
Sp C0	0.8624	0.8624	0.8904	0.9450	0.9450	0.9694	0.9744	0.9744	0.9934
Sp C1	**0.9219**	**0.9219**	**0.9494**	**0.9665**	**0.9665**	**0.9838**	**0.9863**	**0.9863**	**0.9975**
Sp C2	0.9020	0.9020	0.9295	0.9451	0.9451	0.9691	0.9787	0.9787	0.9960
Sp H0	0.8744	0.8908	0.9019	0.9367	0.9533	0.9645	0.9620	0.9757	0.9861
Sp H1	0.8853	0.9005	0.9139	0.9610	0.9684	0.9756	**0.9744**	0.9814	0.9880
Sp H2	**0.9306**	**0.9472**	**0.9558**	**0.9643**	**0.9752**	**0.9843**	0.9704	**0.9828**	**0.9910**

**Table 5:** A comparison of performance evaluation between related works and our approach.

Reference	Technology Device	Acc. (%)	Sp. (%)	Sens. (%)	Classifier	Standard	#level
Our approach	LMC	98 avg			BgT	MDS- UPDRS	0,1,2
Bazgir et al. [49] (2015)	Sony Xperia SP smartphone	91	90.64	89.6	Artificial Neural Network (ANN)	UPDRS	0,1,2,3,4
Rigas et al. [38] (2016)	Wrist-worn sensor	94	-	-	C4.5 Decision Tree	UPDRS	0,1,2,3,4
Jeon et al. [37] (2017)	Wrist-watch type	85.55 (±6.03) ^1^	-	-	Decision Tree	UPDRS	0,1,2,3
							
Bazgir et al. [51] (2018)	Sony Xperia SP Android smartphone	100	-	-	Naive Bayesian	UPDRS	0,1,2,3,4
	STM32F407VG ARM-based microcontroller	94	-	-			
Kim et al. [50] (2018)	Wrist sensor	85	-	-	Convolutional Neural Network (CNN)	UPDRS	0,1,2,3

^1^ The 95% of confidence intervals are provided for accuracy in parenthesis. MDS: Movement Disorder Society; UPDRS: Unified Parkinson’s Disease Rating Scale.
